# Interference Control Modulations Over Conscious Perception

**DOI:** 10.3389/fpsyg.2017.00712

**Published:** 2017-05-10

**Authors:** Itsaso Colás, Mónica Triviño, Ana B. Chica

**Affiliations:** ^1^Laboratorio de Neurociencia Cognitiva, Departamento de Psicología Experimental, Centro de Investigación Mente Cerebro y Comportamiento, Universidad de GranadaGranada, Spain; ^2^Servicio de Neuropsicología, Hospital Universitario San RafaelGranada, Spain

**Keywords:** interference control, conscious perception, error commission, proactive and reactive control, proportion congruent

## Abstract

The relation between attention and consciousness has been a controversial topic over the last decade. Although there seems to be an agreement on their distinction at the functional level, no consensus has been reached about attentional processes being or not necessary for conscious perception. Previous studies have explored the relation of alerting and orienting systems of attention and conscious perception, but the impact of the anterior executive attention system on conscious access remains unexplored. In the present study, we investigated the behavioral interaction between executive attention and conscious perception, testing control mechanisms both at stimulus-level representation and after error commission. We presented a classical Stroop task, manipulating the proportion of congruent and incongruent trials, and analyzed the effect of reactive and proactive control on the conscious perception of near-threshold stimuli. Reactive control elicited under high proportion congruent conditions influenced participants’ decision criterion, whereas proactive control elicited under low proportion congruent conditions was ineffective in modulating conscious perception. In addition, error commission affected both perceptual sensitivity to detect near-threshold information and response criterion. These results suggest that reactivation of task goals through reactive control strategies in conflict situations impacts decision stages of conscious processing, whereas interference control elicited by error commission impacts both perceptual sensitivity and decision stages of conscious processing. We discuss the implications of our results for the gateway hypothesis about attention and consciousness, as they showed that interference control (both at stimulus-level representation and after error commission) can modulate the conscious access of near-threshold stimuli.

## Introduction

The human brain is a complex system capable of processing, integrating, and acting upon an incredible amount of information. In everyday life, we perceive multiple stimuli at the same time, even if we might not be aware of all of them, that is, if we cannot report their perception. In fact, only a limited portion of the information we process becomes part of our conscious experience (see Tononi, 2008 for a review). But what exactly makes that information reportable? Attention has been postulated to act as that gateway for consciousness, enhancing sensory properties of the stimuli to access conscious perception. Numerous experimental studies in brain-damaged patients (Pöppel et al., 1973) and in the healthy population (Bar and Biederman, 1998) have demonstrated that both attended and unattended information can be processed to a certain extent. However, according to the gateway hypothesis (Posner, 2012, 1994), consciousness emerges after the attentional system has filtered out information from our crowded environment (for a review, see Dehaene and Naccache, 2001). This hypothesis considers attentional selection as a necessary although maybe not sufficient condition for consciousness (Chica and Bartolomeo, 2012).

Attention is a complex and heterogeneous system (Posner, 1975; Posner and Petersen, 1990; Petersen and Posner, 2012). In order to better understand how attention modulates consciousness, it is important to analyze the impact that different attention systems can exert on conscious processing. Following Posner and Petersen’s model (Posner and Petersen, 1990; Petersen and Posner, 2012), attention can be dissected into alerting, orienting, and executive control networks. Previous literature has already explored alerting and orienting contributions to conscious perception (Wyart and Tallon-Baudry, 2008; Chica et al., 2010, 2011, 2012, 2016; Kusnir et al., 2011; Wyart et al., 2011; Chica and Bartolomeo, 2012; Botta et al., 2014). For example, Kusnir et al. (2011) found that an auditory cue eliciting phasic alerting improved participants’ ability to discriminate a near-threshold stimulus, especially when targets were temporally unpredictable. Concerning spatial attention, exogenous attention modulates conscious access (Chica et al., 2010, 2011), producing larger (and more consistent) effects than endogenous attention does (Chica et al., 2012). In order to broadly complete the theoretical framework on the relation between attention and conscious perception, modulations of the anterior network of executive control (the third attention network in Posner and Petersen’s model) over consciousness must also be explored.

The executive control network (Posner and Raichle, 1994) refers to a system involved in the voluntary control of processing in novel or complex situations. According to Norman and Shallice’s (1986) model, the executive control system is activated whenever an individual’s acting schema fails to sort out a particular situation. This could happen when the situation is new, complex or dangerous, requires planning or decision making, implies the inhibition of automatic or competing responses, or involves the detection or correction of an error. Although the term executive function has a much broader meaning in psychology (Petersen and Posner, 2012; Diamond, 2013), executive control could be equivalent to its interference component, which includes inhibitory control and interference control. Three core aspects of executive functions can be differentiated: the abovementioned interference control, working memory, and cognitive flexibility or set shifting (Miyake et al., 2000; Lehto et al., 2003; Diamond, 2013). Interference control enables us to selectively attend, focusing on some stimuli or features and suppressing attention to other stimuli.

Previous studies exploring the relation between executive processes and conscious perception have mainly focused on working memory, manipulating its load. High working memory load affects conscious perception, reducing visual processing of attended stimuli, and inducing inattentional blindness (Fougnie and Marois, 2007; Scalf et al., 2011). Active working memory load also influences the attentional blink magnitude (Akyürek et al., 2007), and operation span correlates with the size of the blink (Colzato et al., 2007). Moreover, working memory load has been demonstrated to increase the threshold of subjective visibility, modulating the impact of a prime stimulus on the response to the target (De Loof et al., 2013). Some studies have distinguished between working memory components (executive and visuo-spatial working memory), which differently interact with conscious detection (De Loof et al., 2015). Mental load has also been demonstrated to affect conscious perception. For example, performing an arithmetic cognitive task along with a visual search task produces a decrease of correct responses and an increase of false alarms (Pérez-Moreno et al., 2011). The impairment in visual detection is greater as mental load increases (see also Recarte et al., 2008).

Nonetheless, the above-cited experiments focus either on mental load or on the executive process of working memory, while according to Posner and Petersen’s model of attention (Posner and Petersen, 1990; Petersen and Posner, 2012), the anterior executive network would be more related to interference control. In the present work, we explored whether interference control (a key mechanism of executive attention) would modulate conscious perception, as working memory does.

According to the dual mechanisms of control framework (Braver, 2012), interference control operates via (1) reactive control, which relies upon detection of interference to reactivate task goals; and (2) proactive control, involving sustained active maintenance of task goals. Reactive control suppresses the activation of task-irrelevant information in an online, trial-by-trial basis; whereas proactive control prepares the system, priming task-relevant processing pathways prior to stimulus-onset (De Pisapia and Braver, 2006). Reactive or proactive control mechanisms can be implemented depending on task characteristics. For example, in tasks with high proportion of congruent stimuli (e.g., 75% congruent trials and 25% incongruent trials), participants’ expectancy for interference is low and therefore the most effective control strategy will be to reactivate control mechanisms when an incongruent stimulus appears. In contrast, low proportion congruent tasks (e.g., 25% congruent trials and 75% incongruent trials) induce a high expectancy for interference, making proactive control mechanisms more likely to be recruited (De Pisapia and Braver, 2006; Braver, 2012). Overall, the proactive strategy of control is thought to be more resource consuming. However, on a trial-by-trial basis, incongruent trials will elicit more interference under the reactive control mode than under the proactive control mode, due to the necessity of retrieving inactive goal representations (Braver et al., 2003; Braver, 2012).

In the present research, we explored for the first time in the literature the interactions between interference control and conscious perception. We asked participants to perform a Stroop task along with a conscious detection task, in which participants had to mark the location of a near-threshold target. We analyzed perceptual sensitivity and response criterion to detect the near-threshold stimulus. In order to test the impact of reactive control on conscious perception, we made the proportion of congruent trials larger than the proportion of incongruent trials (75%–25%, respectively, Experiment 1). As a consequence, participants were more likely to recruit reactive control mechanisms when an incongruent stimulus appeared, due to the low expectancy of interference. Our hypothesis was that when facing an incongruent trial in a context of high proportion of congruent trials, the cost of transiently reactivating goal representations would impact conscious perception on that trial (Braver et al., 2003; Braver, 2012). We conducted another experiment (Experiment 2), in which the proportion of incongruent trials was larger than the proportion of congruent trials (75%–25%, respectively). As the implementation of proactive control is thought to involve a sustained maintenance of task-goals along the task, we did not expect any effects of this control mechanism in conscious perception.

Finally, we manipulated timing of control, by presenting the Stroop and conscious detection tasks either in a concurrent (dual task) or sequential procedure. This arrangement allowed us to explore whether interference control would affect conscious perception when presenting the near-threshold stimulus simultaneously with the conflict task or after conflict resolution. Previous evidence suggests that dual tasks involve attentional selection (Sigman and Dehaene, 2008; Tombu et al., 2011) engaging frontal areas common to the executive attention network (Petersen and Posner, 2012). One can expect then that dual tasks should reduce the availability of the executive attention system. Following this idea, we hypothesized that the interference effect would be greater in the concurrent or dual-task procedure, as compared to the sequential procedure.

According to the gateway hypothesis (Posner, 1994, 2012), which considers attention as an important pre-requisite of conscious perception, we expected to observe modulations of perceptual sensitivity and/or response criterion for incongruent trials relative to congruent trials. Following the dual mechanisms of control framework (Braver, 2012), this modulation should be greater in conditions of low interference expectancy (Experiment 1) as compared to conditions of high interference expectancy (Experiment 2), due to the recruitment of reactive control. The effect was expected to be larger under dual-task conditions, i.e., for the concurrent as compared to the sequential task (Sigman and Dehaene, 2008; Tombu et al., 2011). Finally, since detecting and correcting errors is also considered to activate the executive system (Norman and Shallice, 1986), we explored perceptual sensitivity and response bias after Stroop hits and errors, hypothesizing that error commission will impair conscious perception.

## Experiment 1

### Participants

Twenty-three healthy participants from the University of Granada took part in the experiment (3 males, mean age of 21.84 years, SD of 4.03). Data from 22 participants were included in the analyses, as one participant did not finish the experiment. Participants reported to have normal or corrected-to-normal visual acuity, normal color discrimination, no known neurological disorders, and spoke Spanish as their first language. The experiments were approved by the Ethic Committee for Human Participants, University of Granada. All subjects gave written informed consent in compliance with the ethical standards of the 1964 Declaration of Helsinki.

### Apparatus and Stimuli

E-prime software was used to control the presentation of stimuli, timing operations, and behavioral data collection (Schneider et al., 2002). Experiments were conducted using a 24″ screen Intel Computer running at 60Hz. Participants sat at approximately 57 cm from the monitor. Two black markers and a centered fixation point (a black plus sign, 0.5° × 0.5°) were displayed at the beginning of the trial, on a gray color background (49 cd/m^2^). Each marker consisted of a black square outline (7.5° width × 6° height), placed 10° to either the left or the right side of the fixation point. Spanish words for blue (*azul*, 2.5° × 1°), green (*verde*, 3° × 1°), and yellow (*amarillo*, 4.5° × 1°) colors were presented 1° above fixation. Words were presented either in blue, green, or yellow ink, and could make a given trial either congruent (when word meaning and ink color matched) or incongruent (when word meaning and ink color differed). Inside the lateral markers, a Gabor stimulus could appear. Matlab 8.1^1^ was used to create 100 Gabor stimuli (4 cycles/deg. spatial frequency, 2.5° in diameter, SD of 0.3°), with a maximum and minimum Michelson within-stimulus contrast of 0.92 and 0.02, respectively.

### Procedure

**FIGURE 1:**
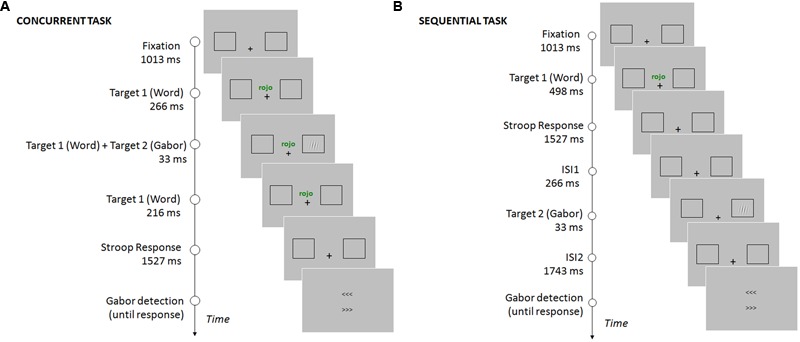
**Timing and sequence of the stimuli on a given trial for the concurrent task (A)** and the sequential task **(B)**. In the concurrent task, the Gabor was presented while the word (Stroop stimulus) was still on the screen. Participants had to report the color of the ink in which the word was written, and then report the location of the Gabor using the arrows (see Procedure). In the sequential task, participants first responded to the word (Stroop stimulus), and the Gabor was presented after the response to the Stroop task was completed. The location of the Gabor was reported using the arrows (see Procedure).

Participants were required to perform two consecutive tasks. First, they had to discriminate the word’s ink color as fast and accurately as possible. Participants responded with their right hand, pressing a keyboard key for each given color (the color-key mapping was counterbalanced across participants, keys “b”, “n”, “m”). In this experiment, stimuli were congruent (the word meaning and ink color matched) on 75% of the trials, and incongruent (the word meaning and ink color did not match) on 25% of the trials. Then, participants performed a Gabor detection task, reporting if they had perceived its appearance. On this task, participants were asked to respond accurately, with no time pressure. They were asked to respond only when they were confident about their perception. The response was given by choosing one of two arrow-like stimuli (> > > or < < <), pointing to the two possible locations of the target: right and left sides of the screen (see **Figure 1**). The arrows were presented one above the other, with their position randomized in each trial. Participants were required to indicate the location of the Gabor with their left hand, pressing an upper keyboard key (“d”) corresponding to the upper arrow, or a lower key (“c”) corresponding to the bottom arrow. This response procedure was employed in order to minimize response preparation and anticipations (Chica et al., 2011). Participants were asked to press the space bar whenever they had not perceived the Gabor stimulus.

Before the experimental trials, Gabor contrast was calibrated for each participant, in the absence of the Stroop stimulus. During titration, participants had to detect the Gabor and select its location. Titration began with a supra-threshold stimulus (Michelson contrast = 0.184), which contrast was manipulated depending on the mean percentage of seen targets every 16 trials. If the participant reported 63% or more targets during the last block of trials, Gabors at the immediately following lower contrast level (Michelson contrast minus 0.009) were used during the next block of trials; besides, if the percentage of seen targets was equal or lower than 38% during the last block of trials, the next block of trials presented Gabors at the immediately following higher contrast level (Michelson contrast plus 0.009). The titration procedure stopped when target contrast yielded a percentage of seen targets > 38% and < 63% for two consecutive blocks of 16 trials.

The experiment was conducted in two separate sessions, each containing titration, practice, and experimental trials. One of the sessions consisted of a concurrent task, while the other consisted of a sequential task (the order of the sessions was counterbalanced across participants). The difference between tasks was the timing of presentation of the stimuli (see **Figure 1**). The experiment consisted of a total of 720 experimental trials divided in two sessions (concurrent and sequential task). Within each session, 270 of the trials were congruent and 90 incongruent. The Gabor was presented in 80% of the trials and absent in 20% of the trials (catch trials). Each session started with 15 practice trials.

### Results and Discussion

We firstly analyzed mean accuracy and reaction times (RTs) to respond to the Stroop task (see **Table 1**). In this experiment, 0.01% of the trials were considered anticipations (RTs faster than 150 ms) and eliminated from the RT analysis. Mean RT and accuracy data were submitted to two independent analyses of variance (ANOVA), with the within participants factors of congruency (congruent and incongruent trials) and task (concurrent and sequential).

**Table 1:** Mean RT and accuracy data for the Stroop task (with standard deviations in parentheses) for congruent and incongruent trials in Experiment 1 (high proportion congruent) and Experiment 2 (low proportion congruent).

			Mean RT, in ms	Mean proportion of correct responses
Experiment 1	Concurrent task	Congruent	682 (154)	0.96 (0.05)
		Incongruent	848 (205)	0.88 (0.06)
	Sequential task	Congruent	611 (149)	0.94 (0.06)
		Incongruent	789 (259)	0.86 (0.08)
Experiment 2	Concurrent task	Congruent	775 (211)	0.93 (0.06)
		Incongruent	821 (209)	0.90 (0.08)
	Sequential task	Congruent	663 (108)	0.94 (0.06)
		Incongruent	706 (110)	0.91 (0.08)

Then, we analyzed responses to the Gabor detection task to explore participants’ conscious perception of the Gabor and its modulation by executive attention (congruent and incongruent trials). We analyzed participants’ responses by using the signal detection theory, which provides a measure of perceptual sensitivity (d′) and response criterion (c). Those indexes were calculated by computing *hits* or correct detections (when participants accurately determined the location of a presented Gabor), *misses* or trials in which the Gabor was presented but participants did not consciously report it, *false alarms* (when participants consciously reported Gabors that were not presented), and *correct rejections* or trials in which the target was not presented and participants reported not having seen it. Trials in which participants incorrectly reported the location of a present Gabor were considered errors and removed from the analyses (1.64% of presented Gabors). Trials in which participants committed an error in the Stroop task were also excluded from the present data analyses (7.17% of the remaining trials). After eliminating Gabor detection errors and Stroop trial errors, a mean of 654 trials (SD = 30) per participant were included in the analyses from Experiment 1.

Perceptual sensitivity (d′) and response criterion (c) were calculated with the following equations: d′ = z(H) - z(FA); c = -0.5^∗^(z(H) + z(FA)). H represents the hit rate, FA represents the false alarm rate, and z corresponds to z-scores, which were calculated using the inverse cumulative distribution function in Microsoft Excel 2011 (NORMSINV). Zero false alarm rates were corrected using the equation proposed by Snodgrass and Corwin (1988): FA = (FA + 0.5)/(FA + CR + 1). For d′, larger values indicate an increased perceptual sensitivity (more hits and/or less false alarms). Concerning the response criterion index, smaller c values indicate a more liberal response criterion (more hits and/or more false alarms), while larger c values imply a more conservative criterion (less hits and/or less false alarms). **Table 2** shows the mean proportion of hits and false alarms for each Stroop condition, task, and experiment.

**Table 2:** Mean proportion of hits and FA (with standard deviations in parentheses) for congruent and incongruent trials in Experiment 1 (high proportion congruent) and Experiment 2 (low proportion congruent), and for Stroop hits and Stroop errors in both experiments.

			Mean proportion of hits	Mean proportion of FA
**Hits and FAs as a function of task and congruency**				
Experiment 1	Concurrent task	Congruent trial	0.60 (0.16)	0.06 (0.07)
		Incongruent trial	0.53 (0.19)	0.06 (0.10)
	Sequential task	Congruent trial	0.50 (0.16)	0.07 (0.08)
		Incongruent trial	0.51 (0.19)	0.07 (0.11)
Experiment 2	Concurrent task	Congruent trial	0.47 (0.19)	0.08 (0.12)
		Incongruent trial	0.46 (0.16)	0.06 (0.08)
	Sequential task	Congruent trial	0.41 (0.18)	0.06 (0.12)
		Incongruent trial	0.39 (0.17)	0.04 (0.07)
**Hits and FAs as a function of task and Stroop accuracy**				
Experiment 1	Concurrent task	Stroop hit	0.55 (0.13)	0.06 (0.07)
		Stroop error	0.42 (0.20)	0.08 (0.23)
	Sequential task	Stroop hit	0.54 (0.16)	0.07 (0.09)
		Stroop error	0.48 (0.21)	0.05 (0.10)
Experiment 2	Concurrent task	Stroop hit	0.47 (0.17)	0.06 (0.08)
		Stroop error	0.44 (0.18)	0.06 (0.12)
	Sequential task	Stroop hit	0.41 (0.17)	0.05 (0.08)
		Stroop error	0.25 (0.20)	0.08 (0.21)

Mean d′ and c indexes were submitted to two repeated measures ANOVA with the within participants factors of congruency and task.

Finally, we analyzed mean d′ and c indexes to detect the Gabor as a function of Stroop accuracy. This analysis was meant to understand whether error commission could alter conscious perception of subsequently presented near-threshold stimuli. We performed two independent ANOVAs for mean d′ and c, with the within participants factors of Stroop response accuracy (Stroop hits and Stroop errors) and task (concurrent and sequential).

For each analysis, participants with mean scores above or below 3 standard deviations (SD) of their group mean were considered outliers and excluded from the analysis. For all analyses, *post hoc* Fisher tests were used to further explore the interactions.

### Stroop Task

After checking for outliers, data from one participant were not included in the Stroop task accuracy analysis. No participants were excluded from the Stroop RT analysis.

When responding to the Stroop task, the expected congruency effect was observed: accuracy was higher for congruent than for incongruent Stroop trials, *F*(1,20) = 44.73, *p* < 0.001, $\eta_{p}^{2}$ = 0.69, and RTs were shorter for congruent than for incongruent trials, *F*(1,21) = 66.16, *p* < 0.001, $\eta_{p}^{2}$ = 0.76. None of the other main effects of interactions reached statistical significance (all *p*s > 0.14).

### Gabor Detection for Congruent and Incongruent Stroop Trials

After checking for outliers, no participants were excluded from the mean d′ analysis. Data from one participant were not included in the mean c analysis.

**FIGURE 2:**
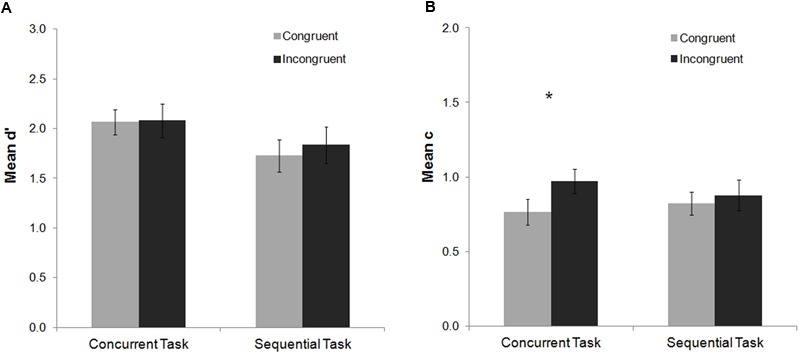
**Mean d′ and c indexes as a function of task and Stroop congruency in Experiment 1.** No significant effects were observed on the analysis of the mean d′ index **(A)**. For the mean c index **(B)** participants maintained a more conservative response criterion for incongruent than congruent trials, especially on the concurrent task, although the interaction between task and congruency was not significant (see Results). Bars represent standard errors. Asterisks represent significant effects for the Fisher *post hoc* comparisons (*p* < 0.05).

The analysis of the mean d′ index did not show any significant main effect or interaction (all *p*s > 0.13), indicating that perceptual sensitivity was not modulated by the factors congruency or task (see **Figure 2**). For the c index, a main effect of congruency was found, *F*(1,20) = 8.61, *p* = 0.008, $\eta_{p}^{2}$ = 0.30, with a more conservative response criterion to detect the Gabor for incongruent than congruent trials (**Figure 2**). Congruency did not interact with task, *F*(1,20) = 2.11, *p* = 0.16, $\eta_{p}^{2}$ = 0.10, although *post hoc* Fisher analyses revealed that the effect was statistically reliable for the concurrent task (*p* = 0.12), and not for the sequential one (*p* = 0.49) (see **Figure 2**). These results indicate that interference control elicited by the Stroop task modulates decisional stages of conscious processing, making participants’ response criterion more conservative for incongruent than congruent trials.

### Gabor Detection After Error and Hit Stroop Trials

After checking for outliers, data from one participant were not included in the mean d′ analysis nor in the mean c analysis of Stroop response accuracy.

**FIGURE 3:**
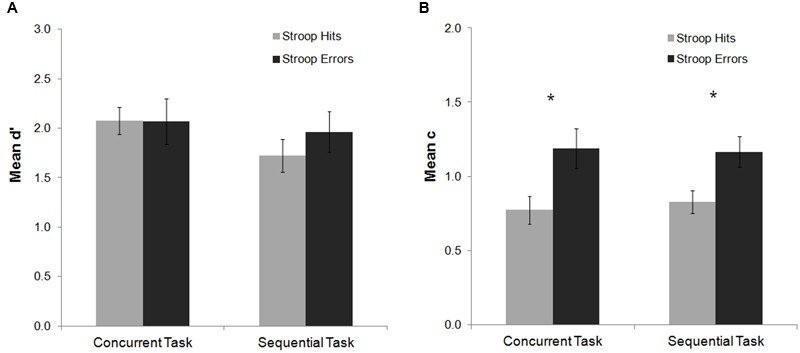
**Mean d′ and c indexes as a function of task and Stroop response accuracy in Experiment 1.** No significant effects were observed on the analysis of the mean d′ index **(A)**. For the c index **(B)** response criterion to detect the Gabor was more conservative after an error on the Stroop task as compared to hit trials for both the concurrent and sequential tasks. Bars represent standard errors. Asterisks represent significant effects for the Fisher *post hoc* comparisons (*p* < 0.05).

For the mean d′ analysis, there were no significant main effects or interactions (all *p*s > 0.25). The analysis of the mean c index showed a main effect of Stroop response accuracy, *F*(1,20) = 20.30, *p* < 0.001, $\eta_{p}^{2}$ = 0.50, indicating a more conservative response criterion to detect the Gabor after Stroop errors as compared to Stroop hits (see **Figure 3**). *Post hoc* Fisher analyses revealed that this effect was statistically significant both for the sequential and the concurrent tasks (both *p*s < 0.001). Thus, committing an error in this experiment did not affect participants’ perceptual sensitivity to detect the Gabor, but instead modulated decisional stages of processing related to response criterion.

## Experiment 2

A second experiment was conducted in order to explore whether results of Experiment 1 could be attributable to the proportion of congruent and incongruent stimuli, rather than to a pure congruency effect. In Experiment 2, we changed the frequency of congruent and incongruent trials in the Stroop task, making the proportion of incongruent stimuli larger than the proportion of congruent stimuli (75 and 25%, respectively). If the observed results were due to stimuli frequency rather than to executive control processes, the inverse patter of result should be observed in Experiment 2, in which incongruent trials were more frequent than congruent trials.

### Participants

A different sample of twenty-three students (2 males, mean age of 20.50 years, *SD* of 1.66) from the University of Granada participated in the experiment.

### Apparatus, Stimuli, and Procedure

Apparatus, stimuli, task, and procedures were the same as Experiment 1 except for the following: we switched the proportion of congruent and incongruent trials to 75% incongruent and 25% congruent trials.

### Results and Discussion

#### Stroop Task

After checking for outliers, data from one participant were excluded from the Stroop task accuracy analysis and from the Stroop RT analysis.

As in the previous experiment, the analyses of mean RTs and accuracy data demonstrated a main effect of congruency. Participants were faster, *F*(1,21) = 62.29, *p* < 0.001, $\eta_{p}^{2}$ = 0.75, and more precise, *F*(1,21) = 9.95, *p* = 0.005, $\eta_{p}^{2}$ = 0.32, for congruent than for incongruent trials. There was also a main effect of task on RTs, *F*(1,21) = 6.80, *p* = 0.016, $\eta_{p}^{2}$ = 0.24, indicating faster RTs in the sequential task than in the concurrent task (see **Table 1**). The interaction between congruency and task was not significant (*F* < 1).

### Gabor Detection for Congruent and Incongruent Stroop Trials

Trials in which participants incorrectly reported the location of a presented Gabor were considered errors and removed from the analyses (1.16% of presented Gabors of Experiment 2). Trials in which participants committed an error on the Stroop task were also excluded from data analyses (8.93% of the remaining trials). After eliminating Gabor detection errors and Stroop trial errors, a mean of 655 trials per participant (SD = 40) were included in the analyses of Experiment 2.

After checking for outliers, no participants were excluded from the mean d′ analysis. Data from one participant were not included in the mean c analysis.

**FIGURE 4:**
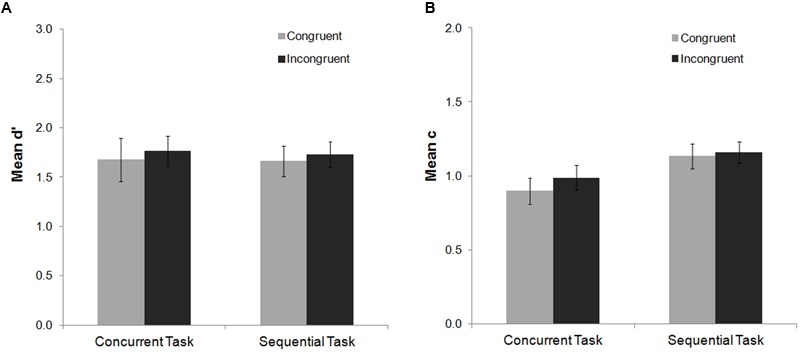
**Mean d′ and c indexes as a function of task and Stroop congruency in Experiment 2.** No main effects or interactions were found for the mean d′ index **(A)**. For the mean c index **(B)** no congruency effects were found. A main effect of task was observed, but it did not interact with the congruency factor. Bars represent standard errors.

The ANOVA of the mean d′ index did not show significant congruency or task effects (*p*s > 0.30), and no significant interaction (*F* < 1) (see **Figure 4**). For the mean c index, a main effect of task was found, *F*(1,21) = 8.05, *p* = 0.01, $\eta_{p}^{2}$ = 0.28, while the main effect of congruency was not significant (*F* = 1). The congruency by task interaction for the mean c index was not significant either (*F* < 1).

In order to directly compare results from Experiment 1 and Experiment 2, we performed two 2-way ANOVAs (one for the concurrent and one for the sequential task). We observed that the congruency effect interacted with Experiment in the concurrent task, *F*(1,41) = 16.36, *p* < 0.001, $\eta_{p}^{2}$ = 0.29, but not in the sequential task, *F* < 1. We also used Bayesian statistics, in which analyses are not biased against the null hypothesis, and we can establish evidence for the absence of an effect only on the observed data. Therefore, with the collected data, we can conclude if the alternative hypothesis is more probable than the null hypothesis or vice-versa. In Bayesian statistics a Bayesian Factor = 1 indicates no evidence in favor of either the null or the alternative (H1) hypothesis. Bayesian Factors < 1 indicate evidence in favor of the null hypothesis (in our case, comparable response criterion for congruent and incongruent trials), while Bayesian Factors > 1 indicate evidence in favor of the H1 hypothesis (in our case, a different response criterion for congruent and incongruent trials). Bayesian Factors > 10 are considered as strong evidence in favor of the H1 hypothesis, while Bayesian Factors > 1 and < 3 indicate anecdotal evidence for H1 (Jeffreys, 1961, cited by Jarosz and Wiley, 2014). A two-tailed repeated-measures Bayesian *t*-test was performed to compare response criterion on congruent and incongruent trials in the concurrent task in Experiments 1 and 2, with the default settings implemented in JASP 0.8.1.1 software (JASP Team, 2016, retrieved from https://jasp-stats.org/) [prior P(H0) = P(H1) = 0.50, Cauchy prior width = 0.707]. Results of the *t*-test performed in Experiment 1 demonstrated strong evidence in favor of the alternative hypothesis (BF_10_ = 15.82). In contrast, the same analyses for the concurrent task in Experiment 2 demonstrated only anecdotal evidence in favor of the alternative hypothesis (BF_10_ = 1.373).

The results of these experiments demonstrate that the effect of congruency found in Experiment 1 for the mean c index was not due to differences on stimulus frequency alone. Low frequency congruent trials did not impact response criterion (Experiment 2) while low frequency incongruent trials did (Experiment 1). Therefore, participants’ response criterion to detect the Gabor on incongruent trials as compared to congruent trials was not more conservative because these trials were less frequent than congruent trials, but because they were both incongruent and infrequent, a known condition to produce a reliable activation of the executive control system, intensifying the interference effect of the Stroop task (Lindsay and Jacoby, 1994). We can therefore conclude that our manipulation of executive attention by the Stroop congruency did not alter perceptual sensitivity, but modulated response criterion under conditions of high executive conflict (Experiment 1), and not merely due to stimuli exposure or stimulus frequency.

### Gabor Detection After Error and Hit Stroop Trials

After checking for outliers, no participants were excluded from the mean d′ analysis of Stroop response accuracy. One participant was not included in the analysis of the mean c index.

**FIGURE 5:**
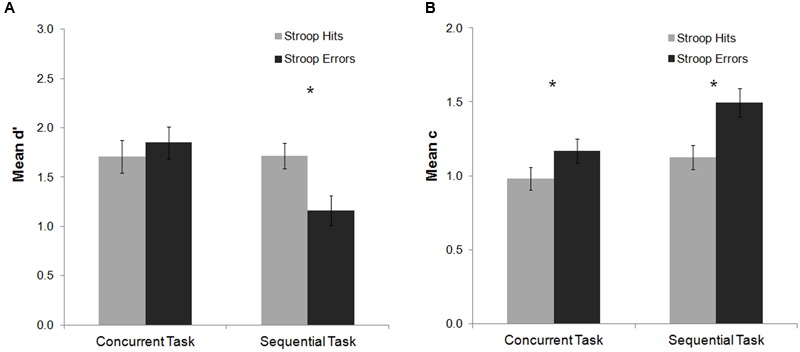
**Mean d′ and c indexes as a function of task and Stroop response accuracy in Experiment 2.** For the d′ index **(A)**, committing an error in the Stroop task impaired participants’ perceptual sensitivity to detect the subsequent Gabor in the sequential task. For the c index **(B)** response criterion to detect the Gabor was more conservative after an error on the Stroop task as compared to hit trials for both the concurrent and the sequential task. Bars represent standard errors. Asterisks represent the significant effects for the Fisher *post hoc* comparisons (*p* < 0.05).

For the mean d′ analysis, a main effect of task was observed, *F*(1,22) = 5.18, *p* = 0.033, $\eta_{p}^{2}$ = 0.19, which was modulated by Stroop response accuracy, *F*(1,22) = 13.82, *p* = 0.001, $\eta_{p}^{2}$ = 0.39. In the sequential task, participants’ ability to detect the subsequent Gabor decreased after Stroop errors as compared to Stroop hits (Fisher *post hoc* analysis, *p* < 0.001). This effect was non-significant for the concurrent task (Fisher *post hoc* analysis, *p* = 0.29) (**Figure 5**). For the mean c analysis, the main effects of Stroop response accuracy and task were significant [*F*(1,21) = 19.31, *p* < 0.001, $\eta_{p}^{2}$ = 0.48; *F*(1,21) = 5.14, *p* = 0.034, $\eta_{p}^{2}$ = 0.20, respectively]. The interaction between both factors was not significant [*F*(1,21) = 2.77, *p* = 0.11, $\eta_{p}^{2}$ = 0.12]. Overall, participants’ response criterion was more conservative after Stroop errors as compared to Stroop hits (both for the concurrent and the sequential tasks, Fisher *post hoc* analysis, both *p*s < 0.02), and for the sequential as compared with the concurrent task.

An unexpected result regarding error commission modulations on conscious perception was the lack of effects in perceptual sensitivity in Experiment 1 (high proportion congruent), as compared to Experiment 2 (low proportion congruent). A plausible explanation for this absence of effect in Experiment 1 could be the difference in Stroop error distribution among experiments. In order to explore whether errors were equally distributed among experiments, we performed an ANOVA on the mean percentage of Stroop errors for participants from the two experiments, with the within-subject factors of congruency and task, and experiment as a between-subject factor. The analysis revealed a significant interaction between congruency and experiment, *F*(1,43) = 14.23, *p* < 0.001, $\eta_{p}^{2}$ = 0.25, indicating that in Experiment 1, participants committed more errors on incongruent Stroop trials compared to congruent trials, while errors in Experiment 2 were more equally distributed among congruent and incongruent trials. I.e., in Experiment 1, most errors were made on incongruent Stroop trials. In Experiment 2, by contrast, the congruency effect was reduced, making this experiment a better condition for observing error commission modulations on conscious perception with no contamination of the congruency factor. Committing an error on the Stroop task in Experiment 1 leaded to a more conservative response criterion to detect the near-threshold stimulus, as incongruent trials from that experiment made participants’ response criterion more conservative. This suggests that the impact of error-commission in Experiment 1 could be masked by the congruency effect, and therefore error commission modulations could be more reliably observed in Experiment 2.

## General Discussion

The present study explored for the first time the interactions between the anterior executive network of attention and conscious perception. In particular, we explored whether inference control would modulate perceptual sensitivity and response criterion to detect near-threshold information. Participants were asked to detect a near-threshold target while performing a classic Stroop task, in a high proportion congruent condition (eliciting reactive control) and in a low proportion congruent condition (eliciting proactive control). In agreement with the gateway hypothesis (Posner, 2012, 1994), if attention were a prerequisite for consciousness, the transient recruitment of non-active task goals under reactive control situations would affect conscious perception, either impairing perceptual sensitivity on incongruent as compared to congruent trials or modulating response criterion. We did not expect modulations of either perceptual sensitivity or response criterion in situations where proactive control was implemented. Moreover, modulations of perceptual sensitivity or response criterion were expected to be larger under dual-task conditions, i.e., for the concurrent as compared to the sequential task. Finally, we predicted that error commission in the Stroop task would impair perceptual sensitivity of the near-threshold stimulus for that given trial or modulate participants’ response criterion in situations in which error commission occurred before the near-threshold stimulus presentation, i.e., in the sequential task. In the concurrent task, by contrast, we did not expect conscious access modulations produced by error commission, because when the Gabor appears, participants have not responded to the Stroop task yet.

As predicted, reactive control mechanisms (elicited under conditions of high proportion of congruent trials) impacted conscious perception, resulting in a more conservative response criterion to report the Gabor on incongruent as compared to congruent Stroop trials. This result could not be accounted for by solely stimulus frequency, as the effect was not observed in the low proportion congruent condition (Experiment 2) (for a review of proportion congruent effects, see Bugg and Crump, 2012).

It could be argued that our results could be explained by working memory load rather than interference control, because in the concurrent task participants had to maintain the response for the Gabor detection task after the Stroop response. Although it is true that working memory requirements are larger in the concurrent task than in the sequential task, working memory requirements are comparable for incongruent trials in both experiments, but these trials differ in their capacity to elicit reactive as compared to proactive control. Therefore, working memory load cannot solely explain the congruency effect reported in the response criterion index.

An alternative or maybe a complementary explanation to our data relates to mental load. Although the concept of mental load is difficult to define, some studies have demonstrated that performing an arithmetic cognitive task along with a visual search task impairs the latter (Pérez-Moreno et al., 2011), and that the higher the mental load of the cognitive task, the greater the impairment in the visual search task. This result is comparable to the congruency effect demonstrated in the present study, but some theoretical differences should be noted. While mental arithmetic tasks largely rely on working memory processes, even if they are not presented within the visual domain, the Stroop task is traditionally associated to interference control rather than to working memory (Miyake et al., 2000). Nonetheless, it is plausible that our results and those found by Recarte et al. (2008) and Pérez-Moreno et al. (2011) are different measures of the same phenomenon.

Finally, some authors could also argue that perceptual load, instead of mental load or interference control, could be mediating our results. In our study, both the Stroop stimuli and the Gabor stimuli were presented in the visual modality, increasing the perceptual load in the concurrent task as compared to the sequential task (Lavie et al., 2014). Although perceptual load is larger in the concurrent task than in the sequential task, it is comparable for congruent and incongruent trials. Following the same logic than above, the congruency effect observed in response criterion was only observed in Experiment 1 (with a larger proportion of congruent trials) and not in Experiment 2 (with a larger proportion of incongruent trials). Perceptual load is comparable for incongruent trials in both experiments, but these trials differ in their capacity to elicit reactive as compared to proactive control. Therefore, perceptual load cannot solely explain the congruency effect observed in the response criterion index.

This new body of research complements the literature on the relation between conscious perception and the different attention networks, broadening our knowledge about how interference control interacts with conscious processing. Particularly, our results support the idea that, as alertness and orienting, executive attention also modulates conscious perception. Importantly, this study confirms that the interference aspect of executive control –and not only working memory load (Fougnie and Marois, 2007; Scalf et al., 2011; De Loof et al., 2013, 2015) or mental load (Recarte et al., 2008; Pérez-Moreno et al., 2011)- affects conscious access of near-threshold information.

Previous research exploring the relation of alerting and orienting systems of attention and conscious perception had demonstrated modulations of perceptual sensitivity by both attentional systems (Wyart and Tallon-Baudry, 2008; Chica et al., 2010, 2011, 2012, 2016; Kusnir et al., 2011; Botta et al., 2014). However, rather than modulating perceptual sensitivity to detect stimuli, interference control impacted participants’ response criterion. These results fit recent literature using the attentional blink phenomenon and working memory encoding, demonstrating that the impairment of the second target detection in this paradigm is due to delayed rather than suppressed processing (Vogel and Luck, 2002; Scalf et al., 2011). Coherent with this idea, the reactivation of behaviorally relevant task goals after conflict detection (reactive control) impacts perceptual decision making rather than modulating perceptual sensitivity. In contrast, recruitment of proactive control, a more efficient strategy, could have actively maintained the representation of both the Stroop task and the conscious detection task goals, preparing the system in a manner that would prevent interference modulations of conscious perception. According to this idea, the interference control aspect of executive attention will influence conscious access in a similar way as working memory load does in inattentional blindness and attentional blink paradigms (Akyürek et al., 2007; Colzato et al., 2007).

Error detection and correction is considered another important function of the executive attentional system (Norman and Shallice, 1986). Moreover, error commission consistently affected the decision criterion to report the near-threshold target, making participants’ criterion more conservative on errors as compared to hit Stroop trials. Contrary to our hypothesis, error commission modulated response criterion not only in the sequential task but also in the concurrent task. This result was unexpected, since in the concurrent task Gabors were presented before participants responded to the Stroop task. However, the Gabor detection response in the concurrent task was given after the Stroop response (and therefore, after Stroop hits or Stroop errors). Hence, it is plausible that participants made the perceptual decision about the Gabor after having responded to the Stroop task in both the concurrent and the sequential tasks. The effect of Stroop response accuracy in the concurrent task could reflect participants’ reinterpretation of their conscious experience after committing an error in the Stroop task (i.e. during the inter-stimulus interval or the Gabor detection response time). Importantly, in the low proportion congruent condition (Experiment 2), committing an error in the sequential Stroop task not only impacted participants’ decision criteria but also modulated perceptual sensitivity in the sequential task, impairing their ability to detect the target when it was presented after a Stroop error as compared to Stroop hits. One possible explanation for this effect could be that, similarly to the post-error slowing phenomenon (Rabbitt, 1966), the impairment to detect near-threshold stimuli after an error could be reflecting performance evaluation processes. In this case, participants would be engaged in apprehending the error-situation, preventing the conscious detection of the subsequent target (Danielmeier and Ullsperger, 2011), as shown by Buzzell et al. (2017). According to the gateway hypothesis, error commission could be preventing the attentional amplification of the near-threshold stimulus, necessary to conscious perception. However, other explanations are possible. For example, both errors on the Stroop task and changes in Gabor detection on a given trial might have been produced by general fluctuations in cognitive control (Leber et al., 2008; Esterman et al., 2013). More research should be done to clarify this issue.

## Conclusion

Results from this study seem to support the gateway hypothesis on the relation between attention and consciousness (Rees and Lavie, 2001; Dehaene et al., 2006; Posner, 1994, 2012). Given that our manipulations of interference control resulted in an impact on the conscious access of near-threshold stimuli, we can conclude that attention acts as a prerequisite for conscious processing, facilitating or preventing a given stimulus from accessing consciousness. However, other models such as the cumulative influence hypothesis (Tallon-Baudry, 2012) suggest independent contributions of attention and consciousness to a single process of perceptual decision-making. According to this hypothesis, attention and consciousness mechanisms separately influence participants’ decision on the perception of a given stimulus. In this model, a decision variable accumulates consciousness-related neural activity, but also, attention-related neural activity. The cumulative influence hypothesis suggests that behavioral reports based on this decision variable could show an interaction between attention and consciousness, whereas neural variables could be separately related to attention and consciousness (Tallon-Baudry, 2012). Future lines of research should attempt to address whether behavioral changes in conscious perception observed in situations of interference control do reflect an actual interaction between attention and consciousness at a neural level.

## Ethics Statement

This study was carried out in accordance with the recommendations of the Ethic Committee for Human Participants, University of Granada, with written informed consent from all subjects. All subjects gave written informed consent in accordance with the Declaration of Helsinki. The protocol was approved by the Ethic Committee for Human Participants, University of Granada.

## Author Contributions

AC conceived and designed the study. IC designed and performed the experiments. AC and IC analyzed and interpreted the data. AC and IC wrote the manuscript that was critically revised by MT. All authors approved the version to be published.

## Conflict of Interest Statement

The authors declare that the research was conducted in the absence of any commercial or financial relationships that could be construed as a potential conflict of interest.

## Footnotes

1 http://www.mathworks.com
